# Morphological and molecular response mechanisms of the root system of different *Hemarthria compressa* species to submergence stress

**DOI:** 10.3389/fpls.2024.1342814

**Published:** 2024-04-04

**Authors:** Bingna Shen, Wenwen Li, Yuqian Zheng, Xiaoli Zhou, Yinuo Zhang, Minghao Qu, Yinchen Wang, Yang Yuan, Kaiyue Pang, Yanlong Feng, Jiahai Wu, Bing Zeng

**Affiliations:** ^1^ College of Animal Science and Technology, Southwest University, Chongqing, China; ^2^ College of Grassland Agriculture, Northwest Agriculture and Forestry University, Shanxi, China; ^3^ Institute of Prataculture, Chongqing Academy of Animal Science, Chongqing, China; ^4^ Institute of Animal Husbandry and Veterinary Medicine, Guizhou Provincial Academy of Agricultural Sciences, Guizhou Key Laboratory of Agricultural Biotechnology, Guizhou, China; ^5^ College of Animal Science and Technology, Southwest University, Chongqing University Herbivore Engineering Research Center, Chongqing, China

**Keywords:** *Hemarthria compressa*, phenylpropanoid biosynthesis, plant hormone signal transduction, molecular mechanisms, transcription factors

## Abstract

**Introduction:**

The severity of flood disasters is increasing due to climate change, resulting in a significant reduction in the yield and quality of forage crops worldwide. This poses a serious threat to the development of agriculture and livestock. *Hemarthria compressa* is an important high-quality forage grass in southern China. In recent years, frequent flooding has caused varying degrees of impacts on *H. compressa* and their ecological environment.

**Methods:**

In this study, we evaluated differences in flooding tolerance between the root systems of the experimental materials GY (Guang Yi, flood-tolerant) and N1291 (N201801291, flood-sensitive). We measured their morphological indexes after 7 d, 14 d, and 21 d of submergence stress and sequenced their transcriptomes at 8 h and 24 h, with 0 h as the control.

**Results:**

During submergence stress, the number of adventitious roots and root length of both GY and N1291 tended to increase, but the overall growth of GY was significantly higher than that of N1291. RNA-seq analysis revealed that 6046 and 7493 DEGs were identified in GY-8h and GY-24h, respectively, and 9198 and 4236 DEGs in N1291-8h and N1291-24h, respectively, compared with the control. The GO and KEGG enrichment analysis results indicated the GO terms mainly enriched among the DEGs were oxidation-reduction process, obsolete peroxidase reaction, and other antioxidant-related terms. The KEGG pathways that were most significantly enriched were phenylpropanoid biosynthesis, plant hormone signal transduction etc. The genes of transcription factor families, such as C2H2, bHLH and bZIP, were highly expressed in the *H. compressa* after submergence, which might be closely related to the submergence adaptive response mechanisms of *H. compressa*.

**Discussion:**

This study provides basic data for analyzing the molecular and morphological mechanisms of *H. compressa* in response to submergence stress, and also provides theoretical support for the subsequent improvement of submergence tolerance traits of *H. compressa*.

## 1 Introduction

Adequate water is the prerequisite and basis for all plant physiological and metabolic activities, and too much or too little water will affect their yield and quality. Influenced by factors such as global warming, extreme precipitation, rising water tables, and irrational irrigation practices, flooding stress has become an important factor limiting plant growth and affecting crop yield and productivity (Zhao et al., 2021). As a major abiotic stress, flooding significantly and negatively affects nearly 16% of global agricultural production (Gao et al., 2022). The effects of flooding stress on plant growth and development mainly involve reactive oxygen ion accumulation, plant photosynthesis and changes in plant sugar content ultimately affecting plant biomass and productivity (Nie G. P. et al., 2021). When plants are subjected to flooding stress, they are unable to maintain normal physiological and biochemical activities due to excessive waterlogging that leads to a hypoxic or anoxic inter-root soil environment. The root system is the first organ to encounter hypoxic stress, which results in significant phenotypic changes, tissue damage, and growth retardation in this environment (Panozzo et al., 2019). The respiration mode of root cells gradually changes from aerobic to anaerobic. The products of anaerobic respiration, ethanol, acetaldehyde, and lactic acid, are accumulated in large quantities in root cells, causing root cell death, reducing the root system vigor, affecting mineral nutrient uptake, and downregulating chlorophyll biosynthesis, leading to a reduction in photosynthesis (Sun et al., 2018; Awan et al., 2023).

To adapt to this unfavorable environment, plants respond to hypoxic stress in three stages. First, plants rapidly induce the production of signal transduction components, leading to the upregulation of a wide range of stress-responsive genes, which regulate the plant glycolysis and fermentation pathway for energy supply and the antioxidant defense system facilitating reactive oxygen species (ROS) scavenging. Lastly, morphological adaptations occur to improve plant resilience through the formation of aerated tissues, adventitious roots, and other anatomical adaptations (Voesenek and Bailey-Serres, 2013; Loreti et al., 2016; Fukao et al., 2019). In recent years, the studies on the response mechanism of plants to flooding stress and other abiotic stress has continuously increased, with transcriptomics characterization being one of the most frequently implemented approaches. A complex regulatory network for plant flooding tolerance has been identified in model plants such as Arabidopsis (*Arabidopsis thaliana*) and rice (*Oryza sativa*), where metabolically regulated pathways and phytohormone signaling pathways are interconnected (Licausi et al., 2011). Arabidopsis plants subjected to flooding stress are accompanied by rapid systemic physiological and transcriptomic responses that involve ROS, calcium, and hydraulic waves responses, as well as the induction of hypoxic adaptation mechanisms in systemic tissues (Peláez-Vico et al., 2023). A large number of differentially expressed genes, including many well-known hypoxia-induced genes, such as those encoding glycolytic and fermentative enzymes have been identified in the roots of kale-type oilseed rape (*Brassica napus*) varieties after 4 and 24 h of hypoxia (Ambros et al., 2022), which is very similar to the hypoxia response of the model organism Arabidopsis. Involvement of phytohormones ethylene, abscisic acid (ABA) and gibberellin (GA) in the regulation of rice elongation growth response under waterlogging stress (Xiong et al., 2013). The *Sub1* gene, an ethylene response factor, is the most intensively studied gene for flooding tolerance, and the true function of this gene is to inhibit energy metabolism in rice by suppressing GA synthesis, thereby inhibiting the massive consumption of energy for growth. At the same time, this gene also has the function of inhibiting chlorophyll degradation. Figuratively speaking, this gene can make rice “dormant” and green under flooding, and is the core gene in the waterlogging tolerance pathway (Wang S. C. et al., 2016). A study identified a key gene, *OsUGT75A* (encoding a UDP-glucosyltransferase), that controls the rapid elongation of rice germ sheaths under flooded conditions. This gene precisely regulates the free ABA and JA contents in seeds and germ sheaths through glycosylation of abscisic acid (ABA) and jasmonic acid (JA), thereby mediating the elongation of germ sheaths through the ABA and JA signaling pathways, which further reveals the molecular mechanism of rice flooding tolerance through the “escape strategy” (He et al., 2023). Transcriptomic analysis of root tissues of flood-tolerant and flood-sensitive orchardgrass (*Dactylis glomerata*) cultivars, following submergence stress, identified DEGs of phytohormone-related genes, mitogen-activated protein kinases (MAPK), and Ca^2+^ signaling genes that were significantly differentially expressed in submergence-tolerant orchardgrass (Qu et al., 2023). Although the importance of research on the response mechanisms to crop flooding stress has been recognized, due to the wide range of variability between plant species and habitats, their adaptation mechanisms to flooding stress also vary significantly. Therefore, in-depth studies on the response mechanisms of different genotypes of each species under flooding stress are vital for developing superior varieties with higher tolerance to flooding.

Transcription factors are upstream in the regulatory network of plant growth and development, influencing the expression of multiple genes in the plant and are able to respond to specific stress conditions, which in turn allows for differential expression of proteins (Zhang et al., 2019). Involved in a variety of biological processes such as plant growth, development and immune (Song et al., 2022). *Sub1a* belongs to the ERFVII transcription factor genes that regulate ERFVII protein activation to transcribe hypoxia-responsive genes during plant hypoxia (Loreti and Perata, 2023). In the model plant Arabidopsis thaliana, the ERFVII transcription factor was found to promote the establishment of an ethylene-negatively regulated AR system, which inhibits the growth of the primary root system and promotes AR elongation in response to hypoxic stress (Eysholdt-Derzsó and Sauter, 2019). bZIP family of TFs has important roles in regulating plant growth, development and abiotic stress (Van et al., 2016). The extremely flood-tolerant strong bean variety Qi Huang 34 was subjected to flooding, and root tissue samples were subjected to transcriptome sequencing, which led to the isolation and identification of the gene encoding the bZIP transcription factor *Glyma04g04170*, which is differentially expressed in response to flooding tolerance in combination with AREB/ABFb (Lin et al., 2020). AP2/ERF transcription factors play an important role in plant tolerance to flooding hypoxia (Xie et al., 2022). Zhang et al. used kiwifruit (*Actinidia* sp.) as experimental material for transcriptomic analysis after 4 d of flooding treatment and identified TFs of the AP2/ERF, WRKY, TGA, MYB, and bZIP families, of which 14 up-regulated and 14 down-regulated genes were identified in the AP2/ERF family of TFs, suggesting that these genes play a role in the response to flooding in kiwifruit (Zhang et al., 2015).

*H. compressa* is a perennial high-quality forage grass of the Poaceae family with excellent quality, high regrowth capacity, and adaptability, which is widely planted and applied in southern China (Li X. L. et al., 2019). In addition, *H. compressa* is one of the most economically and ecologically significant forage crops due to its tall plant type, large leaf volume, and good palatability by cattle, sheep, and rabbits, which is favorable to ecological environment construction and animal husbandry development. It is advantageous in terms of water and nutrient access, resistance to environmental stresses, and fast growth (Lin et al., 2019). However, the research on *H. compressa* in abiotic stress is still limited. At present, research on *H. compressa* resistance has mainly focused on drought resistance (Yang et al., 2013; Jin et al., 2011), cold resistance (Zhang and He, 2018), metal resistance (Chen et al., 2011; He et al., 2015) and other aspects. The molecular research on *H. compressa* mainly concentrates on molecular marker development, which has played an important role in studying *H. compressa* genetic diversity, variety selection, and gene localization (Nie J. R. et al., 2021). However, there are only a few reports on transcriptome studies of *H. compressa*, as genome research in this species is of high complexity and high workload, but the implementation of high-throughput sequencing technologies can be implemented to obtain almost all transcripts from tissues or cells (Li et al., 2010). Recently, a multi-omics database platform was established (Milletdb, http://milletdb.novogene.com/), which consists mainly of legumes (Sun et al., 2023). However, this genome still needs to be augmented with grasses to further understand their evolutionary history and to find more resistance candidate genes for improved breeding. Therefore, using transcriptome sequencing and others to analyze and understand the molecular response mechanism of *H. compressa* under submergence stress can further elucidate its response to stress and provide a theoretical basis for selecting and breeding superior varieties in the future.

In view of this, the present study was conducted to investigate the gene expression and molecular mechanisms under submergence stress in the root systems of two *H. compressa* genotypes with different submergence stress tolerance, namely, the flood-tolerant GY and the flood-sensitive N1291, by using RNA-seq. These results will provide a theoretical basis for the further study of the flood tolerance mechanisms of *H. compressa.*

## 2 Materials and methods

### 2.1 Plant material cultivation and treatments

In this experiment, two cultivars of *H. compressa* with large differences in flooding tolerance screened out before were selected as test materials, namely, the flood-tolerant GY and the flood-sensitive *H. compressa* N1291. GY was obtained from the College of Grassland Science and Technology of Sichuan Agricultural University, China, and N1291 from Shimiao Village, Xinli Town, ZhongXian County, Chongqing City, China. The pot test method was adopted, using cuttings. Six plants were planted per pot, and 50 pots were prepared for each cultivar. The soil used in the pots was containing vermiculite, vegetative soil, and perlite (3:1:1, v/v/v). The *H. compressa* cultivars were grown in an incubator with a humidity of 70-85%, temperature of 22/15°C (day/night), photoperiod 14 h/10 h (day/night), and 5000 lux light intensity. The plants were irrigated with 1/2 Hoagland nutrient solution twice a week. The submergence stress experiment was carried out when the plants had grown to a 15-20 cm height and were in the tillering stage. Vigorous and uniform *H. compressa* plants were selected for the stress treatment, and the plants were placed into a bucket of water for submergence. The top of the plant leaves was always submerged during the treatment. Root morphology indicators include root length and root number. Root length and number were measured and photographed at 0 d, 7 d, 14 d, and 21 d after submergence stress, and each treatment was repeated three times. Root samples were taken at 0 h, 8 h, and 24 h after submergence stress, frozen immediately in liquid nitrogen, and stored at -80°C for subsequent analyses. Each treatment was repeated three times, with a total of 18 samples collected. The GY control group was named GY-0h, the submerged treatment groups were named GY-8h and GY-24h, the N1291 control group was named N1291-0h, the submerged treatment groups were named N1291-8h and N1291-24h.

### 2.2 RNA extraction, sequencing, transcript splicing

Total RNA was extracted from the root tissues of *H. compressa* that were submerged under water for 0, 8, and 24 h according to the instructions of RNA prep Pure Plant Total RNA Extraction Kit (Tian gen Biochemical Technology, Beijing, China). Then, the samples were evaluated for RNA concentration, purity, and integrity using an Agilent 2100 bioanalyzer (Agilent, USA). The samples that passed the RNA quality and quantity control were used for the construction of cDNA libraries. After the libraries were constructed, they were preliminarily quantified using a Qubit 2.0 Fluorometer and then diluted to a 1.5 ng/ul concentration. Then, the insert size in the library was detected using an Agilent 2100 bioanalyzer. qRT-PCR was subsequently used to accurately quantify the library’s effective concentration to ensure the library’s quality. Sequencing was performed on the Illumina Nova Seq 6000 (Illumina, USA). Since there is currently no reference genome for *H. compressa*, a *de novo* assembly of the clean reads was used as a reference sequence. Trinity was utilized to assemble the clean reads to resolve problems caused by splicing variations, and all the samples were integrated and assembled, thus making the assembly results more accurate (Grabherr et al., 2011). The Unigene assembly obtained was used as the reference sequence, and the clean reads were mapped on the reference sequence. Transcript level comparison was made using the RSEM software after removing the reads with low-quality values, those that did not form a comparison pair, and those that were aligned to multiple regions of the genome (Li and Dewey, 2011). In this study, read count data were used and normalized by DESeq2 for differential analysis. The differential expression results of each comparison were analyzed using DESeq2 software. Fold change values were calculated using read count averages. All reads have been placed in the Serial Read Archive (SRA) under the login number PRJNA1053919. GO and KEGG enrichment analysis was performed on the differentially expressed genes identified. GO enrichment analysis was performed by GO seq (Young et al., 2010), and KEGG functional enrichment analysis was performed on the differentially expressed gene sets using the KOBAS software. |Log_2_ fold-change (ratio of gene expression between two samples) | > 1 and *P*-value (Padj) < 0.05 were the standard for screening differentially expressed genes. After GO and KEGG enrichment analysis, the genes related to submergence tolerance pathways were identified as key candidate submergence tolerance genes.

### 2.3 Validation of qRT-PCR

Quantitative real-time fluorescence PCR (qRT-PCR) was used to validate the transcriptome sequencing results. Six genes were randomly selected for validation. 18s RNA was used as the internal reference gene, and primers were designed using Primer 5.0 (**Supplementary Table S1**). The samples were first reverse transcribed from RNA to cDNA according to the instructions of the Prime Script™ RT reagent Kit with gDNA Eraser (TaKaRa) reagent. Then, qRT-PCR was performed in the following steps: pre-denaturation at 95°C for 30 s, followed by 40 cycles of denaturation at 95°C for 10 s, and annealing and extension at 60°C for 30 s. The relative expression was calculated by the 2^-ΔΔCT^ method.

## 3 Results

### 3.1 Changes in root morphology after submergence stress

**Figure 1:**
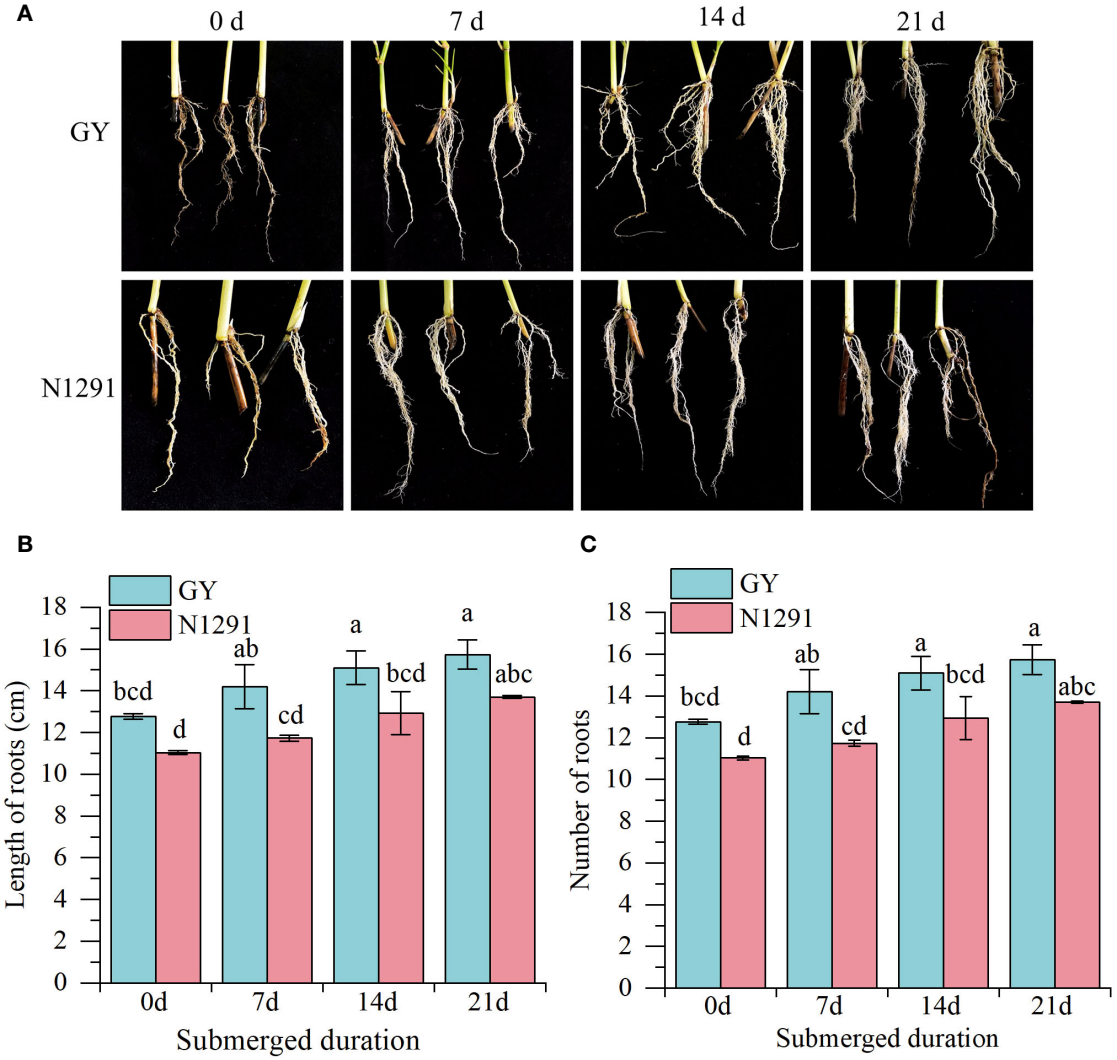
Effects of submergence stress on root morphology of *H. compressa*. **(A–C)** The phenotype of GY and N1291 at 0 d, 7 d, 14 d and 21 d **(A)**. Effects of submergence on the root lengths and numbers of GY and N1291. Error bars indicate the standard error, n=3. Different letters indicate the significant differences. Statistical analysis using one-way analysis of variance (ANOVA) using Duncan’s multiple range test (*P*<0.05).

The root morphology of two *H. compressa* species differed considerably after submergence stress. The length of the adventitious root of the *H. compressa* GY and N1291 cultivars exhibited an increasing trend with the prolongation of submergence stress (**Figure 1A**). The root lengths of *H. compressa* GY were significantly higher than those of the control group by 2.33 cm and 2.97 cm at 14 and 21 d, respectively(*P*<0.05). N1291 increased by 2.63 cm at 21 d of stress compared to the control, the difference was significant (*P*<0.05). The root length of GY was longer than that of N1291 throughout the flooding treatment, being significantly higher (*P*<0.05) at 7 and 14 d (**Figure 1B**). The number of adventitious roots of both *H. compressa* cultivars showed an increasing trend during the stress period (**Figure 1C**). N1291 exhibited a nonsignificant difference and a smaller increase than GY during the whole stress treatment compared with the control.

### 3.2 Data analysis of RNA-Seq and transcript splicing

**Figure 2:**
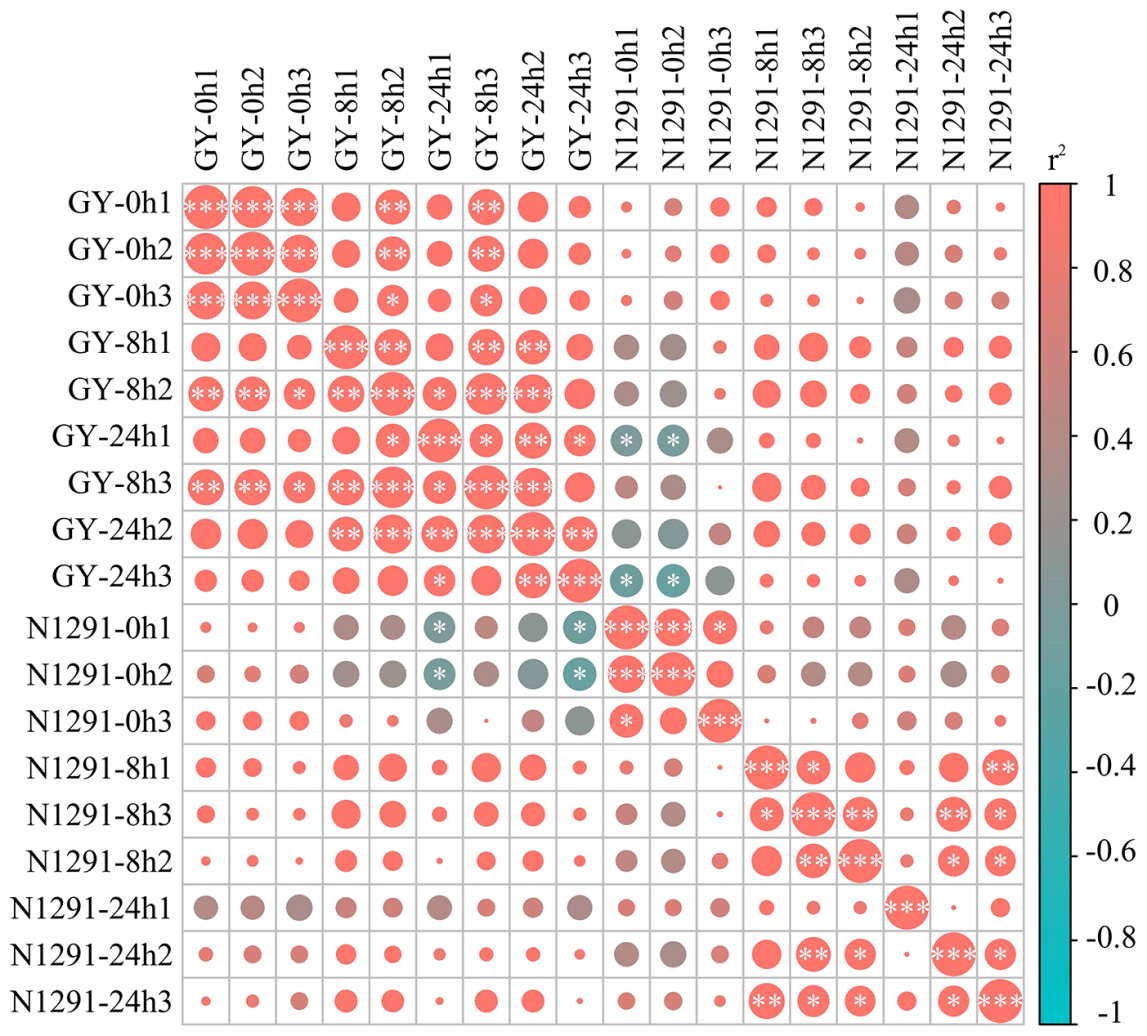
Sample correlation diagram. Based on 18 samples from GY and N1291 (root samples from 0h, 8h and 24h of submergence stress, with three replicates taken for each sample), correlations between each treatment group were determined using a hierarchical clustering approach. Correlation coefficients were calculated by Pearson, with red color representing positive correlation and blue color representing negative correlation, and *, ** and *** denoting *p*<0.05, *p*<0.01 and *p*<0.001, respectively.

After sequencing and filtering out low-quality reads and reads containing N bases, 393,344,027 clean reads, and 118.10 Gb clean bases were obtained. The Q20 percentage of sequences from each sample was above 96.42%, and the error rate was less than 0.03%. The Q30 percentage was above 91.12%, and the GC content was above 53.42% (**Supplementary Table S2**). Thus, the sequencing results of the 18 samples were highly reliable, and the r^2^ among biological replicates was above 0.8 (**Figure 2**). Therefore, the RNA-seq data were reliable and could be used for subsequent analysis. Principal component analysis was subsequently performed, with the three samples within each group clustering together while the samples between the groups were dispersed. All three samples from GY and N1291 in the same period of submergence stress were clustered together (**Supplementary Figure S1**), while the samples from the different submergence stress time points at 8 h and 24 h were clustered distantly compared to the control group at 0 h. This indicates that the reproducibility within each treatment group was relatively good, and there was a significant difference in the expression patterns of samples between different subgroups, which could be further analyzed. The *H. compressa* transcripts lengths were mostly in the range of 300-500 bp. The total number of transcripts was 504,139, with an average length of 1045 bp, an N50 length of 1,515 bp, and an N90 length of 446 bp. Unigene exon length was mostly concentrated in the 300-500 bp range, and the total number of Unigenes was 256,550, with an average length of 862 bp. The average length was 862 bp, the N50 length was 1138 bp, and the N90 length was 396 bp (**Supplementary Table S3**). The N50 values of both transcripts and Unigenes exceeded their respective average lengths, indicating that the transcriptome assembly’s quality and integrity were high, satisfying the requirements of the subsequent functional annotation and other analyses.

### 3.3 Screening for differentially expressed genes

**Figure 3:**
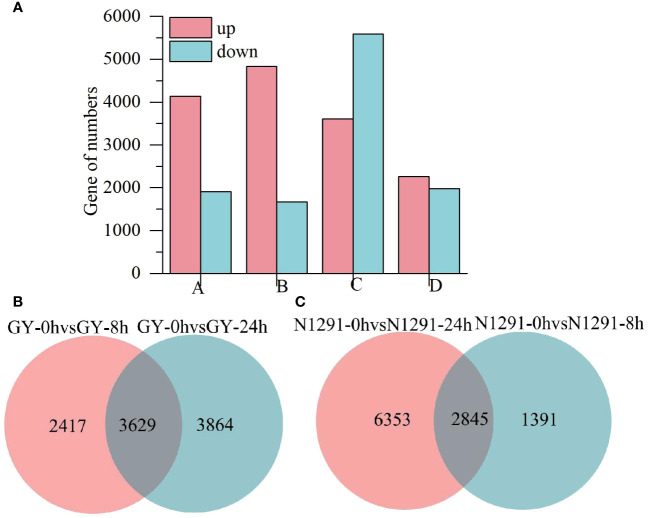
Differentially expressed gene statistics **(A)**, the A represents GY-0h vs GY-8h, B represents GY-0h vs GY-24h, C represents N1291-0h vs N1291-8h, D represents N1291-0h vs N1291-24h. The Venn diagram of differential genes **(B, C)**.

To investigate the DEGs in GY and N1291 in response to submergence stress, the DEGs of the two *H. compressa* varieties were screened with a |log_2_ fold-change| >1 and Padj < 0.05 as the threshold. Compared with 0 h, a total of 6,046 DEGs (4,137 up-regulated and 1,909 down-regulated) were detected at 8 h of submergence stress and 7493 DEGs (4828 up-regulated and 2665 down-regulated) at 24 h of submergence stress in GY. While in N1291, a total of 9198 DEGs (3606 up-regulated and 5592 down-regulated) and 4236 DEGs (2261 up-regulated and 1975 down-regulated) were detected at 8 h and 24 h of submergence stress, respectively (**Figure 3A**; **Supplementary Table S4**). Comparison of GY-0h vs GY-8h and GY-0h vs GY-24h under submergence stress revealed a total of 3629 DEGs identified in both 8 h and 24 h during the 24 h period of stress. Moreover, the comparison of N1291-0h vs N1291-8h and N1291-0h vs N1291-24h under submergence stress revealed 2845 DEGs identified in both 8 h and 24 h of stress (**Figures 3B, C**). The above results indicated that GY exhibited a greater number of differentially expressed genes than N1291 under submergence stress and that GY had more differentially expressed genes at 24 h of stress than at 8 h. On the other hand, N1291 had a greater number of differentially expressed genes at 8 h of stress than at 24 h, suggesting a difference between the two in their responses to submergence stress.

### 3.4 GO and KEGG antioxidant-related pathway enrichment

**Figure 4:**
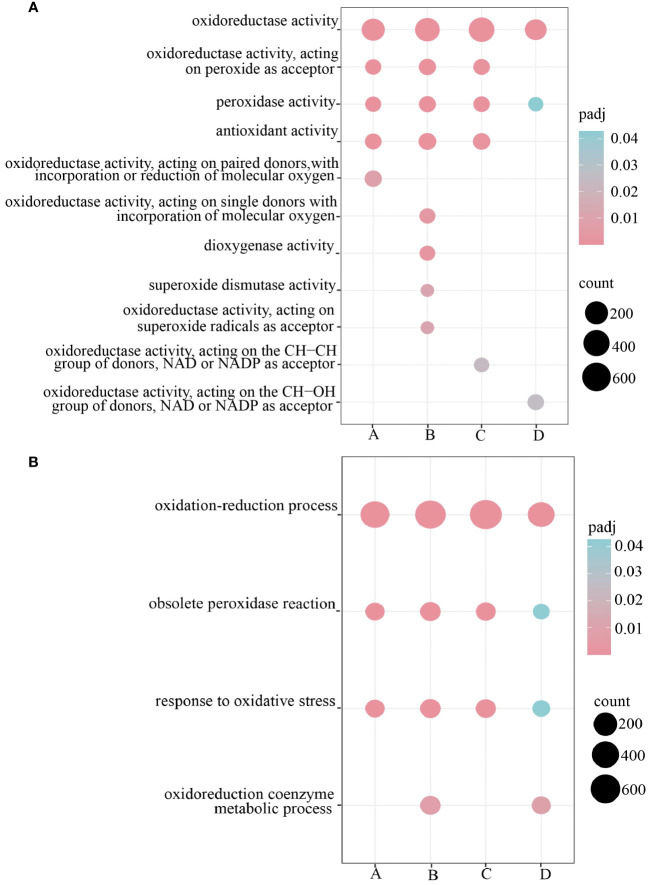
Antioxidant-related pathway GO was enriched and significantly enriched only in MF **(A)** and BP **(B)**, A represents GY-0h vs GY-8h, B represents GY-0h vs GY-24h, C represents N1291-0h vs N1291-8h, D represents N1291-0h vs N1291-24h.

To further investigate the functions of DEGs in the root system of *H. compressa* under submergence stress, GO and KEGG enrichment analyses were performed on the DEGs between GY and N1291 at different time points under submergence stress, respectively. The DEGs in both GY and N1291 groups were assigned to the biological processes (BP), cellular components (CC), and molecular functions (MF) GO categories. This suggests that the root system of *H. compressa* can adapt to hypoxic and anoxic conditions under flooding and submergence stress through metabolism, induction of various enzyme activities, and transport proteins. As shown in **Figures 4A, B** (**Supplementary Table S5**), demonstrating the DEG enrichment in antioxidant enzyme-related pathways in GY and N1291, 2,733 DEGs were significantly enriched in 4 biological processes related to antioxidant enzymes, including oxidation-reduction process, obsolete peroxidase reaction, response to oxidative stress and oxidoreduction coenzyme metabolic process. Moreover, 3,053 DEGs were significantly enriched in 11 molecular functions categories associated with antioxidant enzymes, including oxidoreductase activity, oxidoreductase activity, acting on peroxide as acceptor, peroxidase activity, and antioxidant activity metabolic processes.

**Figure 5:**
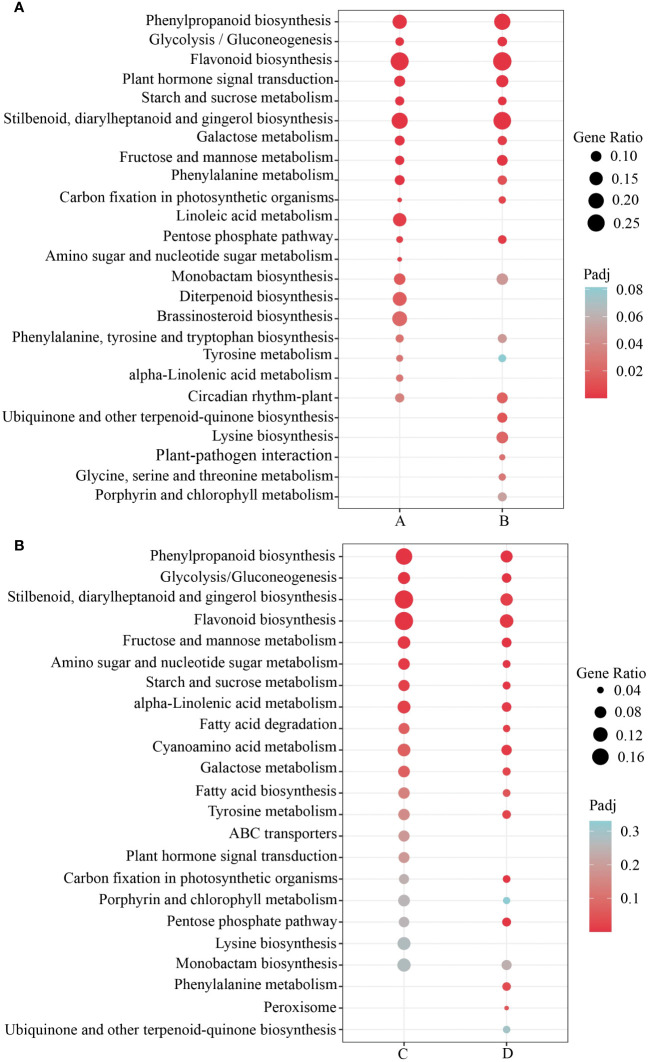
KEGG enrichment analysis of GY **(A)** and N1291 **(B)** groups. A represents GY-0h vs GY-8h, B represents GY-0h vs GY-24h, C represents N1291-0h vs N1291-8h, and D represents N1291-0h vs N1291-24h.

To investigate the key pathways involved in the differentially expressed genes in response to submergence stress in *H. compressa*, 119 signaling pathways were identified to beat both 8 and 24 h of submergence stress in GY, while 119 and 110 signaling pathways were enriched in N1291, respectively. KEGG enrichment analysis was performed on the differentially expressed genes between the two cultivars at different time points of submergence stress (**Supplementary Table S6**). The top 20 most enriched KEGG pathways were selected for further analysis. The differentially expressed genes between GY and N1291 at 8 and 24 h were enriched in the antioxidant-related pathways such as phenylpropanoid biosynthesis, glycolysis/gluconeogenesis, flavonoid biosynthesis, plant hormone signal transduction, starch and sucrose metabolism and other antioxidant-related pathways, suggesting that *H*. *compressa* may recruit antioxidant defense mechanisms through these pathways, removing reactive oxygen species to defend against hypoxic or anoxic environments caused by submergence stress (**Figures 5A, B**). Notably, plant hormone signal transduction and phenylalanine metabolism were significantly enriched in the GY group but not in the N1291 group. Therefore, it can be speculated that these pathways and genes may be related to the increased flooding tolerance of GY.

#### 3.4.1 Analysis of phenylpropanoid biosynthesis in the root system of *H. compressa* under submergence stress

**Figure 6:**
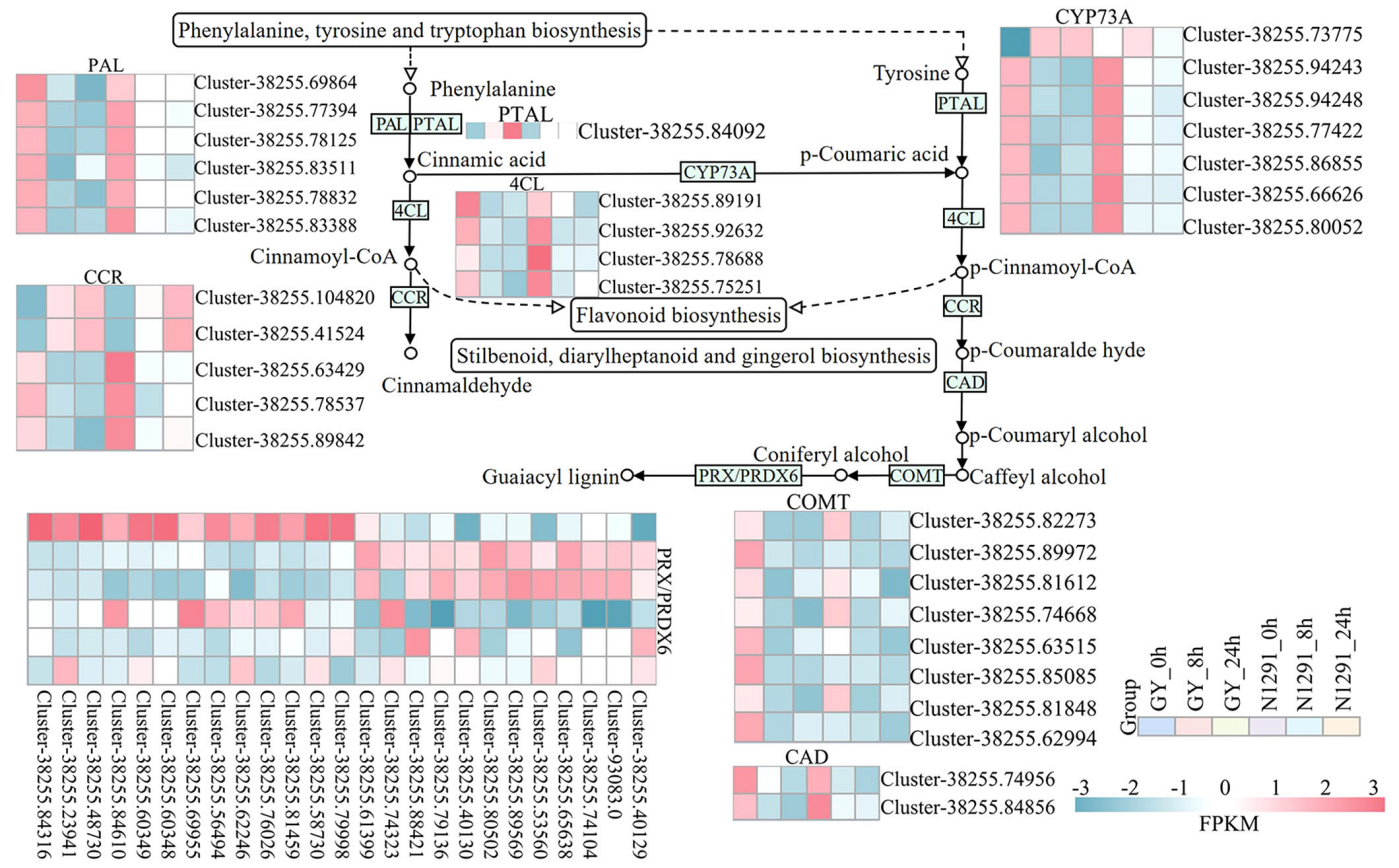
Partial phenylacetone biosynthetic pathway. The y-axis shows the cluster dendrogram of DEGs, and the x-axis shows the sample groups.

In this study, DEGs identified between the *H. compressa* GY and N1291 cultivars were significantly enriched in the phenylpropanoid biosynthesis pathway at both 8 and 24 h of submergence (**Figure 6**), suggesting that this pathway plays an important role in *H. compressa* responses to submergence stress. In this pathway, phenylalanine is converted to cinnamic acid by the deamidation by phenylalanine ammonia-lyase (PAL) and phenylalanine/tyrosine ammonia-lyase (PTAL). Cinnamaldehyde is synthesized from related secondary metabolites in the biosynthetic pathway, such as plant 4-coumarate-CoA ligase (4CL) and cinnamoyl-CoA reductase (CCR). Annotation of the differentially expressed genes revealed nine PAL genes and four 4CL genes that were down-regulated in the submergence treatments between GY and N1291 at 8 and 24 h and up-regulated at 0 h. One PTAL gene was up-regulated in GY at 8 and 24 h of submergence treatment and down-regulated in N1291 at all three treatment time points. In addition, five CCR-related genes were down-regulated except for *Cluster-38255.104820* and *Cluster-38255.41524*, which were up-regulated at 8 and 24 h. Trans-cinnamate 4-monooxygenase (CYP73A) is a cytochrome A member of the P450 monooxygenase superfamily (P450s) and is involved in synthesizing many polyphenolic compounds, such as flavonoids and lignans (Zhu et al., 2020). In the present study, seven CYP73A genes were identified as DEGs, which play an important role in the synthesis of lignin and a range of flavonoids, promoting the synthesis of p-Coumaric acid from Cinnamic acid, an important flavonoid antioxidant. Among them, *Cluster-38255.73775* was up-regulated at GY-8h, 24 h, and N1291-8h and down-regulated at other time points. In contrast, the remaining six genes were down-regulated in all treatment groups in both cultivars. Cinnamyl-alcohol dehydrogenase (CAD) and caffeic acid 3-O-methyltransferase/acetyl serotonin O-methyltransferase (COMT) are the key enzymes involved in the synthesis of lignin, where the downstream product of CAD, p-Coumaryl alcohol is an important flavonoid, and the downstream product of COMT, lignin monomer coniferylalco-ho1, generates guaiacyl lignin under the catalytic activity of peroxidases. CAD genes were all down-regulated in expression at 8 and 24 h of submergence treatment in both varieties. Moreover, there were large differences in the expression patterns between GY and N1291 in COMT and PRX/PRDX6-related genes, but most of the genes showed a trend of down-regulation of expression during the submergence stress. The above results suggest that the expression of various antioxidant-related genes is altered in the *H. compressa* root system in response to submergence stress. In addition, the expression of lignin synthase-related genes was down-regulated to reduce lignin biosynthesis to maintain the root structure, reduce the lignification rate, and maintain normal physiological activities. This shows that the phenylpropanoid biosynthesis pathway may play an important role in plant adaptation and tolerance mechanisms under submergence stress.

#### 3.4.2 Analysis of plant hormone signal transduction in the root system of *H. compressa* under submergence stress

**Figure 7:**
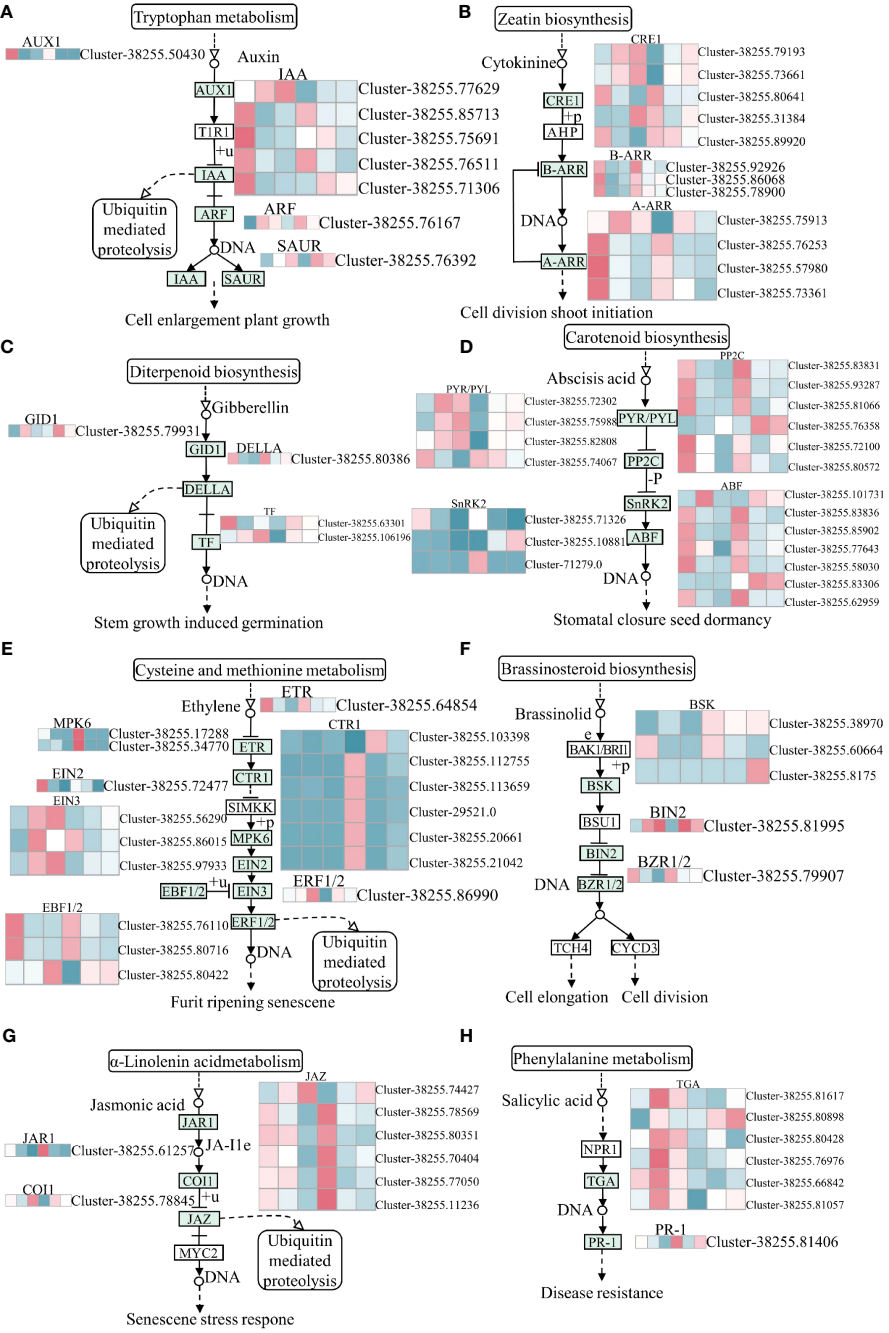
Plant hormone signal transduction pathway. **(A)** Auxin, **(B)** Cytokinine, **(C)** Gibberrllin, **(D)** Abscisis acid, **(E)** Ethylene, **(F)** Brassinolide, **(G)** Abscisic acid, **(H)** Salicylic acid biosynthesis pathway. The y-axis shows the cluster dendrogram of DEGs, and the x-axis shows the sample groups.

In the present study, plant hormone signal transduction pathways were significantly enriched in the GY group but not in N1291 after submergence stress, which might be the reason for the difference in their adaptation to submergence stress. Submergence stress-activated various plant hormone signaling pathways in the roots of *H. compressa*, namely auxin, cytokinin (CTK), gibberellin (GA), abscisic acid (ABA), ethylene (ETH), brassinosteroids (BR), jasmonic acid (JA), and salicylic acid (SA) (**Figure 7**). In this study, auxin response factor (ARF), auxin-responsive protein IAA, auxin influx carrier (AUX1), and SAUR family protein were among the auxin pathway genes involved in submergence stress. Among those, significant differences were observed in the expression patterns of IAA genes in GY and N1291, with most of the genes down-regulated during treatment, while they were significantly induced in GY. The AUX1 gene was down-regulated at 8 and 24 h in both varieties, whereas the ARF and SAUR were up-regulated, suggesting that the latter were activated under submergence stress. The CTK signaling pathway involves the two-component response regulator ARR-A family (A-ARR), cytokinin receptor (CRE1), and two-component response regulator ARR-B family (B-ARR), of which five CRE1 genes were significantly induced in GY at 8 and 24 h of submergence stress. In contrast, five CRE1 genes were significantly induced at 8 and 24 h in N1291. Among the two-component response regulator ARR-B family (B-ARR), The B-ARR genes were down-regulated at 8 and 24 h of stress in GY and up-regulated at 0 h in GY and N1291. However, they were not significantly expressed in both treatment groups of N1291. The four A-ARR genes were consistently up-regulated in expression at 8 and 24 h of GY stress, except for *Cluster-38255.75913*, which was expressed to a higher extent at 8 h than at 24 h, whereas it was not significantly expressed at 0 h. (**Figure 7**). Thus, based on our results, these genes were repressed under submergence stress. Several genes, including the abscisic acid receptor PYR/PYL family (PYR/PYL), ethylene-insensitive protein 3 (EIN3), the brassinosteroid insensitive 2 (BIN2) protein, and the SA responsive TGA transcription factor was up-regulated 8 and 24 h of submergence stress in GY and N1291, indicating that these genes were activated under submergence stress. Taken together, these results revealed that most DEGs were down-regulated in expression after submergence stress. Therefore, we speculate that submergence stress may inhibit phytohormone signal transduction, and its inhibitory effect increases with increasing submergence time.

### 3.5 Identification of transcription factors

Transcriptional regulation is one of the major strategies for plant adaptation to adversity stress, and transcription factors are key proteins regulating the spatial and temporal expression of downstream target genes (Wu et al., 2022). In the stress network signaling pathway, transcription factors are involved in regulating plant growth and development and stress responses by binding cis-acting elements to repress or activate the downstream expression of certain genes (Strader et al., 2022). Using Plant TFDB (a plant transcription factor database), 5666 transcription factors were annotated to the *H. compressa* transcriptome database. They belonged to 92 transcription factor families, and the top 5 transcription factor families with the highest number of members identified were: C2H2, zn-clus, bZIP, and bHLH families (**Supplementary Figure S2**; **Supplementary Table S7**). The expression of these five important transcription factors was altered at different points of submergence stress (**Table 1**). It is noteworthy that C2H2 transcription factors were the most numerous among the TFs identified. Zinc finger proteins (ZFPs) form a transcription factor family, among which the most studied belong to C2H2-type zinc finger proteins. Studies have shown that the zinc finger protein gene family plays an important role in eukaryotic growth and development and in response to adversity stress. Many transcription factor families contain zinc finger structures, which are tightly associated with adversity stress response regulation (Zhong et al., 2020). In this study, after submergence stress, there were differences in the expression of C2H2 transcription factors between the GY and N1291 treatment groups at 8 and 24h of submergence stress. 80 and 72 C2H2 transcription factors were differentially expressed genes in the GY treatment group, respectively, while 175 and 138 genes were in the N1291 group, respectively. Thus, the DEGs that encoded for C2H2 transcription factors in N1291 were greater in number compared to GY. We speculate that the possible reason is that N1291 is poorly tolerant to flooding and can initiate the expression of more stress-associated transcription factors, especially under short-term submergence stress.

**Table 1:** Statistics on the number of differential genes of important transcription factors.

Transcription factors	Comparison group	DEGs number	Up-DEGs	Down-DEGs
C2H2	GY-0h vs GY-8h	80	55	25
	GY-0h vs GY-24h	72	46	26
	N1291-0h vs N1291-8h	175	103	72
	N1291-0h vs N1291-24h	138	100	38
zn-clus	GY-0h vs GY-8h	24	21	3
	GY-0h vs GY-24h	26	20	6
	N1291-0h vs N1291-8h	85	66	19
	N1291-0h vs N1291-24h	45	36	9
*bHLH*	GY-0h vs GY-8h	46	27	19
	GY-0h vs GY-24h	53	29	24
	N1291-0h vs N1291-8h	59	41	18
	N1291-0h vs N1291-24h	46	29	17
bZIP	GY-0h vs GY-8h	40	24	16
	GY-0h vs GY-24h	39	21	18
	N1291-0h vs N1291-8h	70	31	39
	N1291-0h vs N1291-24h	51	28	23

### 3.6 Real-time fluorescence quantitative PCR validation

**Figure 8:**
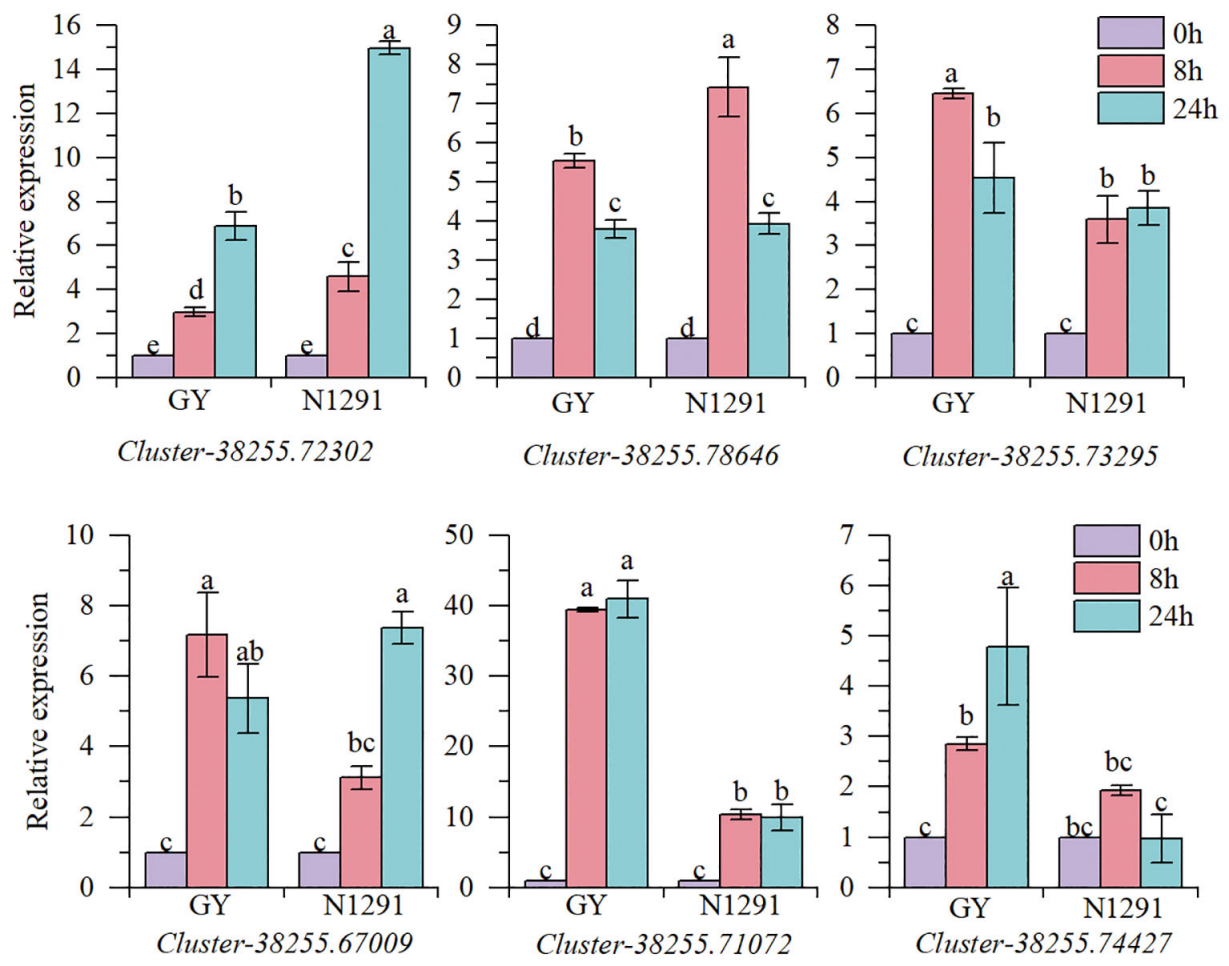
Validation of genes expression using qRT-PCR. Error bars indicate the standard error, n = 3. Different letters indicate the significant differences. Statistical analysis using one-way analysis of variance (ANOVA) using Duncan’s multiple range test (*P*< 0.05).

In this experiment, six genes related to the submergence stress adaptation in *H. compressa* were selected to verify the reliability of the transcriptome results by fluorescence quantitative PCR test. The results are shown in **Figure 8**. The genes *Cluster-38255.74427* and *Cluster-38255.72302* are involved in the phytohormone signal transduction pathway, *Cluster-38255.78646* and *Cluster-38255.73295* are involved in the starch and sucrose metabolic pathway, *Cluster-38255.67009* and *Cluster-38255.71072* are associated with the glycolysis/glycolysis pathway. The expression trends of the six genes in the fluorescence quantitative PCR were consistent with the transcriptome results, indicating that the transcriptome sequencing data in this study were highly accurate and reliable.

## 4 Discussion

### 4.1 Submergence stress promotes the formation of a large number of adventitious roots in *H. compressa*

Morphological characteristics of the root system after flooding stress can be a direct response to its growth status. Energy deficiency caused by hypoxia due to the flooded environment leads to a reduction in nutrient uptake by the plant, resulting in a cessation of root elongation, leading to significant root damage and death of the root tip (Htwe et al., 2019). Flooding stress stimulates the growth of adventitious roots, which are an important means of adaptation or resistance to flooding in many plants, and the ability of adventitious root cells to divide and their physiological activity is enhanced under flooding stress, thus maintaining normal physiological activities of transportation and oxygen acquisition (Wei and Li, 2000). In deep-water rice, elongation of ARs under partial flooding stress began after 8 to 10 h, and ethylene promoted the formation of ARs by triggering the death of epidermal cells covering root tips (Mergemann and Sauter, 2000). In this study, submergence stress induced ethylene production, which triggered the trend of increasing adventitious root length and root number of both *H. compressa* GY and N1291 during submergence stress, which shows that under submergence stress, *H. compressa* can increase the surface area of the root system by elongating the adventitious roots and producing ARs to enhance the transportation and uptake of oxygen, which is a proactive way of submergence resistance to take a shelter from hypoxia. The results of this study were similar to those of Wei et al, which showed that *H. compressa* could respond to the stress by rapidly producing adventitious roots and elongating the root system at the early stage of flooding, but at the later stage of flooding, the more resistant species had stronger adaptive ability than the less resistant ones, and they could regulate the growth of the root system longer and stably in order to adapt to and withstand the stress, which could reduce the damage of the submergence stress. The root growth of more resistant varieties in the stage of submergence was more adaptive than that of less resistant varieties (Wei et al., 2013; Phukan et al., 2014). In the present study, the number of adventitious roots increased with submergence stress in both *H. compressa* cultivars., However, the increase in GY was more pronounced compared to N1291, which may be a manifestation and a reason for the differences in flooding tolerance between the cultivars.

### 4.2 Response of antioxidant-related mechanisms after submergence stress

Energy metabolism and the antioxidant defense system are the main mechanisms employed by plants to adapt to short-term submergence stress, which puts plant tissues in a low-oxygen or anaerobic state. Mitochondrial respiration and oxidative phosphorylation are blocked, and the plants experience a significant energy crisis (Zhou et al., 2020). Plants initiate a series of metabolic activities to supply energy and reduce its dissipation to resist submergence injury to maintain energy supply in their tissues (Schmitz et al., 2013). In this study, we found that the DEGs of two cultivars of *H. compressa* under submergence stress were significantly enriched in pathways related to the oxidation-reduction process, obsolete peroxidase reaction, carbohydrate metabolic process, tetrapyrrole binding, heme binding, oxidoreductase activity, and catalytic activity. All of these metabolic pathways are related to energy metabolism and the antioxidant system. Therefore, the synthesis and metabolism of intracellular molecules are important in *H. compressa* adaptation and tolerance to submergence stress. In addition, carbohydrates are an important source of energy metabolism in plants, which exist as structural and non-structural forms, with lignin being one of the important components of structural carbohydrates (Shan et al., 2021). Changes in the quantity and composition of lignin and the accumulation of lignin pathway intermediates and derivatives potentially altered the transcript levels of genes involved in the submergence stress response. Changes in lignin structure affect its physicochemical properties and may have a negative impact on plant growth, whereas some phenylpropanoid pathway intermediates have been shown to inhibit phenylpropanoid pathway enzymes (Vanholme et al., 2019). The DEGs in the root systems of the two *H. compressa* cultivars in this study were mostly involved in phenylpropanoid biosynthesis and the lignin catabolism pathway, whereas the related genes regulating key enzymes of lignin synthesis, such as PAL, CYP73A, 4CL, COMT, and CAD, were significantly down-regulated under submergence stress. We speculate that this is because photosynthesis is significantly reduced under submergence stress in the above-ground tissues of *H. compressa*. The root system mainly produces energy from anaerobic respiration to maintain life activities. The carbon utilization rate of the anaerobic respiration pathway is much lower than that of aerobic respiration (Qiao et al., 2020), which may lead to a greater proportion of phosphoenolpyruvic acid participating in the glycolysis pathway instead of the phenylalanine-producing *H. compressa* pathway which is the precursor for the synthesis of lignin, moreover, lignin biosynthesis is an energy-consuming and irreversible process (Vogt, 2010). Therefore, this may be one of the energy and carbon-saving strategies adopted by the *H. compressa* root system as a short-term response to submergence stress by down-regulating the expression of lignin synthase-related genes, affecting lignin biosynthesis. Similarly, a study demonstrated that the down-regulated expression of phenylpropanoid biosynthesis genes in sesame (*Sesamum indicum*) root system significantly slowed down secondary metabolites production after plants were subjected to flooding stress (Wang L.H. et al., 2016). In addition, our study found that phenylpropanoid biosynthesis, glycolysis/gluconeogenesis, and flavonoid biosynthesis pathway genes were significantly enriched in both *H. compressa* cultivars under submergence stress. It suggested that these metabolic pathways are involved in the maintenance of energy metabolism homeostasis in the root system of *H*. *compressa*.

**Figure 9:**
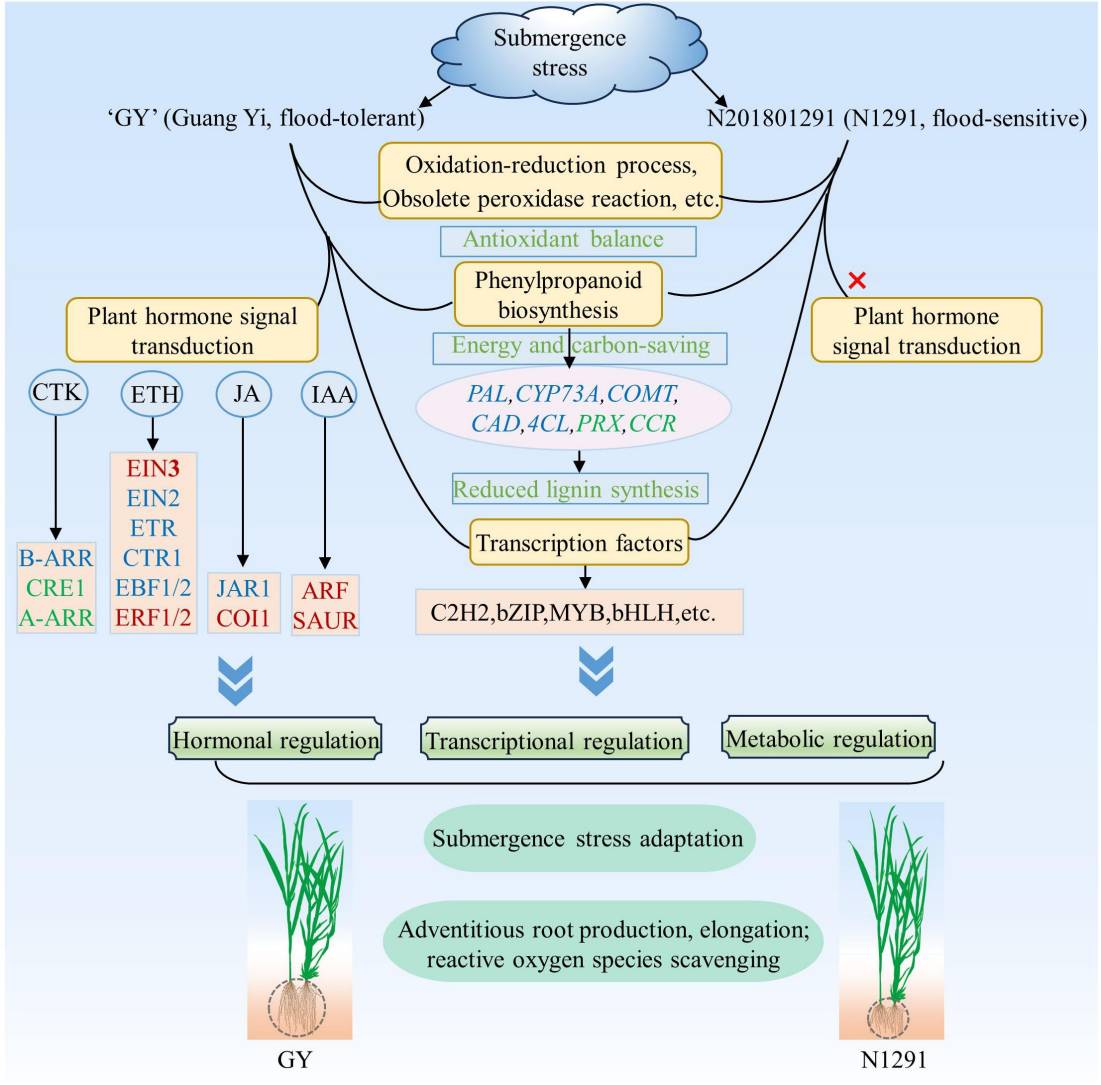
Proposed modeling of antioxidant mechanisms in *H. compressa* under submergence stress. Red font indicates that the gene or transcription factor was up-regulated during stress, green font indicates that the gene or transcription factor was down-regulated, and blue font indicates that there was a change. The ovals represent genes and the rectangles represent transcription factors. *H. compressa* responded to submergence stress by hormonal regulation, metabolic regulation and transcriptional regulation through a series of antioxidant pathways, plant hormone signal transduction, phenylpropanoid biosynthesis pathway and transcription factors. However, N1291 did not respond to stress through the plant hormone signal transduction.

Phytohormones are important plant growth and development regulators under biotic and abiotic stresses. Plant adaptation and tolerance to abiotic stresses require complex sensing, signaling, and stress response mechanisms. Plants can respond to damage caused by external stresses in plants by regulating the levels of hormones in the plant body (Waadt et al., 2022). Phytohormones play the role of signaling molecules in regulating plant response to abiotic stresses. The increased ethylene levels in plants under flooding stress demonstrate its key regulatory functions during the early response to flooding stress. It has been shown that the interaction of ethylene with other hormones, such as ABA, GA, IAA, and CTK, is involved in plant responses and adaptation to hypoxic or anaerobic conditions to attain flooding stress tolerance (Zhao et al., 2018). Key components of the ethylene signaling pathway have been identified, including ETR1, CTR1, EIN2, and EIN3/EIN3-LIKE (EIL) transcription factors. Their signaling activates the expression of ethylene-responsive genes through EIN3/EIL1-mediated transcription to trigger the ethylene response (Perata, 2020). Ethylene induces the expression of genes regulating GA synthesis, increases GA content in plants, accelerates internode elongation, and allows plants to escape from hypoxic environment (Hattori et al., 2009). BR can interact with GA under the regulation of the *Sub1A* gene to limit the aboveground elongation of rice. The DELLA family members and the GA signaling repressor SLR1 protein can negatively regulate GA-mediated responses that inhibit rice plants’ elongation under flooding conditions (Lin et al., 2004). In addition, these reports indicated that the production of these phytohormones can induce the development of many ARs to enhance the plant’s functions further, with the involvement of ABA-related signaling pathways. Thus, phytohormone metabolic pathways are associated with the production of ARs under flooding conditions. This also suggests that the elongation and formation of ARs of *H. compressa* in this study may be related to the production of ethylene, but unfortunately we did not find obvious elongation and increase in the number of *H. compressa* roots when we observed the root system at 8 h and 24 h of submergence, and this phenomenon was more obvious at 7 d, 14 d, and 21 d of submergence, so it is not difficult to speculate that ethylene and other plant hormones play a positive role in this, but it may also be be related to its strong tolerance to submergence. For example, in *Arabidopsis*, it was found that ethylene can act with NO to regulate the downward biased growth of its leaves, and it can also be involved with reactive oxygen species in regulating or inducing the formation of ventilated tissues in *Arabidopsis* to promote the uptake and utilization of O_2_ as well as the transport of O_2_ after exposure to flooded water (Hebelstrup et al., 2012). ABA plays a key role in plant adaptation to various environmental stresses (Qi et al., 2018). PP2Cs are one of the core modules of ABA signaling. It appears in the early stage of ABA signaling, when ABA deficiency stimulates PP2C production, inhibits SnRK2s activating enzyme activity, and vice versa inactivates PP2C (Gong et al., 2020). In this study, plant hormone signal transduction was significantly enriched in GY but not in N1291, which may be related to the increased submergence tolerance of GY compared to N1291, the up-regulation of EIN3 and ERF1/2-related genes in the ethylene-related pathway in the GY cultivar, the down-regulation of GID1 and DELLA, and the up-regulation of PYR/PYL suppressed the downstream expression of PP2C, SnRK2, ABF and ABA regulatory genes under submergence stress. The production of endogenous hormones in plants promotes the transport of IAA. When the accumulation of IAA reaches a certain extent, it will induce adventitious root production, thus promoting adaptation to flooding stress (Pan et al., 2021). This suggests that N1291 does not have that pathway to respond positively to submergence stress, which results in it being less resistant to submergence stress than GY. A study showed that sparsely flowered water hyacinth branches escaped flooding stress by decreasing cytokinin content, consistent with the results of this study (Chen et al., 2022). In addition, some researchers found that *CsEIN3* can exist as a signaling molecule in the ethylene signaling response pathway under flooded environment, thus inducing reactive oxygen species signaling in the root system, which ultimately manifests itself in promoting the production of adventitious roots of cucumber (*Cucumis sativus* L.) in response to waterlogging stress under flooded environment (Xu et al., 2018). Under flooding, ethylene can mediate the production of rhizogenic mechanosignals in rice root tissues, whereas reactive oxygen species can induce programmed cell death in root epidermal cells, yet ethylene and reactive oxygen species can co-regulate the production of ARs in the root system (Steffens et al., 2012). Therefore, we hypothesized that *H. compressa* could initiate the expression of a series of phytohormone-related genes to scavenge or synergize reactive oxygen species under submergence stress through the phytohormone signaling pathway, which collectively enhances its water tolerance through a number of antioxidant-related mechanisms. However, the performance of this ability under prolonged submergence needs to be further investigated. In this study, a simple response model was constructed to explain the response mechanisms of *H. compressa* under submergence stress, such as hormonal regulation and transcriptional regulation, by comparing the submergence tolerance mechanisms of the submergence-tolerant variety GY and the sensitive variety N1291 (**Figure 9**).

### 4.3 Response of transcription factors

Transcription factors (TFs) are a class of proteins that can bind to DNA on specific sequences and regulate gene expression, and TFs play a crucial role in most processes that occur in organisms (Blanc-Mathieu et al., 2023). TFs are central regulators of plant response to abiotic stress (Sun et al., 2020; Yan et al., 2023). Many TF families, including bHLH, C2H2, WRKY, MYB, and GATA, have been shown to be involved in plant stress response by activating stress-responsive gene expression (Ma et al., 2022; Yin et al., 2023). In this study, many TFs were regulated by submergence stress (**Table 1**), and these TFs may contribute to the flooding resistance of *H. compressa*. C2H2 proteins are the main TFs that regulate plant responses to various stresses and plant development. Currently, most of the reports on C2H2-type zinc finger proteins have been focused on drought, low temperature, salt treatment and other adversity stresses, and few reports have been made on flooding stresses (Huang et al., 2020). Specific activation of bZIP, C2H2, TIFY and WRKY by inoculation of arbuscular mycorrhizal fungi (AMF) under flooding stress helps to alleviate flooding stress injury to reed (*Phragmites australis*) (Ban et al., 2023). bHLH14 regulates hormone signaling such as ethylene, ABA and JA against flooding stress (Liu et al., 2023). AtMYB44 in *Arabidopsis* activates the scavenging system and inhibits ROS production (Gu et al., 2019). MYB TFs are considered to be the second largest TF family responding to flooding stress, and it has been reported that MYB TFs play a key regulatory role in flooding stress in many plants, and genes for WRKY and MYB are induced to be expressed under flooding conditions and are highly responsive (Zhang et al., 2022). Among the *Arabidopsis* bZIP gene family, *AtbZIP2* can respond to a variety of stresses such as drought, heat, low temperature, and high salt (Weltmeier et al., 2009), which led to the hypothesis that the bZIP gene family may be a key transcription factor for the stress tolerance of *H. compressa.* However, there is a lack of studies on the function of this transcription factor in submergence stress, and the related functions are broad and lack validation. Under flooding stress, WRKY33 can activate the expression of the hypoxia-responsive gene RAP2.2 to positively regulate the hypoxia response, and the phosphorylated form of WRKY33 plays an important role in plant flooding tolerance, and phosphorylated WRKY33 enhances the activation of RAP2.2 to enhance the tolerance to flooding stress (Vadde, 2021). MYC transcription factors are important members of the bHLH family, and recent studies have shown that MYC2 transcription factors are important components of a hormone regulatory network centered on jasmonic acid (JA), which is involved in the regulation of oxidative stress response in plants and interacts directly with phytohormones such as JA, ABA and GA (Li G. et al., 2019). In this study, we screened four families of TFs with high numbers of C2H2, zn-clus, bZIP, and bHLH members, which may play important roles in *H. compressa*. root system response to submergence. In addition, most of the genes related to the transcription factor family of bHLH, bZIP and C2H2 were up-regulated and expressed after submergence stress in this study, which suggests that submergence stress induces their expression, but further characterization and gene function validation need to be carried out. Among them, the C2H2-type transcription factor family was annotated as high as 876 and the number of up-regulated genes was high after submergence stress, which showed that this transcription factor was induced in response to submergence stress and thus played an important role in the response to submergence stress (**Supplementary Figure S2**). In this paper, only the expression patterns of relevant transcription factors were counted, however, there was no focus on the identification of a particular transcription factor gene family and the expression patterns of its specific genes to clarify its specific function. Members of transcription factors such as bHLH, bZIP and C2H2 responded more strongly to submergence stress and participated in the expression regulation at different time points, which may be a complex process, and may lay a theoretical and practical foundation for the subsequent in-depth study of the function of transcription factor genes in *H. compressa*, as well as provide a reference for the breeding of *H. compressa* resistance.

## 5 Conclusion

In this study, the root morphology and transcriptome of *H. compressa* were analyzed under different times of submergence threat. The results showed that the submergence threat could increase the length and number of adventitious roots of *H. compressa* significant differential gene expression was observed in *H. compressa* root system after 8 and 24 h of submergence. Differential gene function enrichment analysis indicated that Phenylpropanoid biosynthesis, Flavonoid biosynthesis and other pathways were the important regulatory pathways for *H. compressa* GY and N1291 to resist submergence stress, but the flooding-resistant GY responded to submergence stress through the phytohormone signaling pathway more significantly than the flooding-poorly resistant N1291. C2H2, Others, zn-clus, bZIP, and bHLH are important transcription factors for *H. compressa* root system to resist submergence stress. In this study, the molecular mechanism of *H. compressa* root system in response to submergence stress was preliminarily explained, and data support was provided for further research on the functions of submergence tolerance genes in *H. compressa*.

## Data availability statement

The datasets presented in this study can be found in online repositories. The names of the repository/repositories and accession number(s) can be found below: PRJNA1053919 (SRA).

## Author contributions

BS: Data curation, Writing – original draft. WL: Data curation, Writing – original draft. YuZ: Investigation, Writing – review & editing. XZ: Writing – review & editing. YiZ: Data curation, Writing – review & editing. MQ: Writing – review & editing. YW: Writing – review & editing. YY: Writing – review & editing. KP: Data curation, Writing – review & editing. YF: Data curation, Writing – review & editing. JW: Writing – review & editing. BZ: Writing – review & editing.
