# Supplementary material for: Morphological and molecular response mechanisms of the root system of different Hemarthria compressa species to submergence stress
Source: Front Plant Sci. 2024 Apr 4;15:1342814. doi: 10.3389/fpls.2024.1342814 (PMC11024365; doi:10.3389/fpls.2024.1342814)
Supplement: Supplementary file 1 [file Table_2.docx]

Supplementary Table S1. Target gene primer list table

| Gene Id | Forward primer（5'-3'） | Reverse primer（5'-3'） |
| --- | --- | --- |
| *Cluster-38255.74427* | CCATCAGGCTTTCACCAT | CACCGAACCGTTGTAGAA |
| *Cluster-38255.72302* | CAAGAAATTCCACCGCACAC | CTGGCTGCTTGTCCTAGTTAG |
| *Cluster-38255.78646* | TCTGATTACTTCGCTGGTGTC | TCAACCGTATGACCTCCAATG |
| *Cluster-38255.73295* | GAATGGCGGTTGAACAAT | CCTGACTGGTGTATATGGAT |
| *Cluster-38255.67099* | AGTCCCGTTCTCCTCAAG | GCTCGCCATCAAGTACAC |
| *Cluster-38255.71072* | GCTGCTGTCAAAGTTAAGG | TGTCTTCAGACGAGGAATG |
| 18s RNA | CAACCATAAACGATGCCGA | AGCCTTGCGACCATACTCC |
