# Supplementary material for: Morphological and molecular response mechanisms of the root system of different Hemarthria compressa species to submergence stress
Source: Front Plant Sci. 2024 Apr 4;15:1342814. doi: 10.3389/fpls.2024.1342814 (PMC11024365; doi:10.3389/fpls.2024.1342814)
Supplement: Supplementary file 3 [file Table_4.docx]

Supplementary Table S2. Statistics of transcript splicing

| Index | 300-500bp | 500-1kbp | 1k-2kbp | Mean length | N50（bp） | N90（bp） |
| --- | --- | --- | --- | --- | --- | --- |
| transcripts | 177717 | 152612 | 109751 | 1045 | 1515 | 446 |
| Unigenes | 112017 | 81872 | 42090 | 862 | 1138 | 396 |
