# Supplementary figures and images for: Morphological and molecular response mechanisms of the root system of different Hemarthria compressa species to submergence stress
Source: Front Plant Sci. 2024 Apr 4;15:1342814. doi: 10.3389/fpls.2024.1342814 (PMC11024365; doi:10.3389/fpls.2024.1342814)

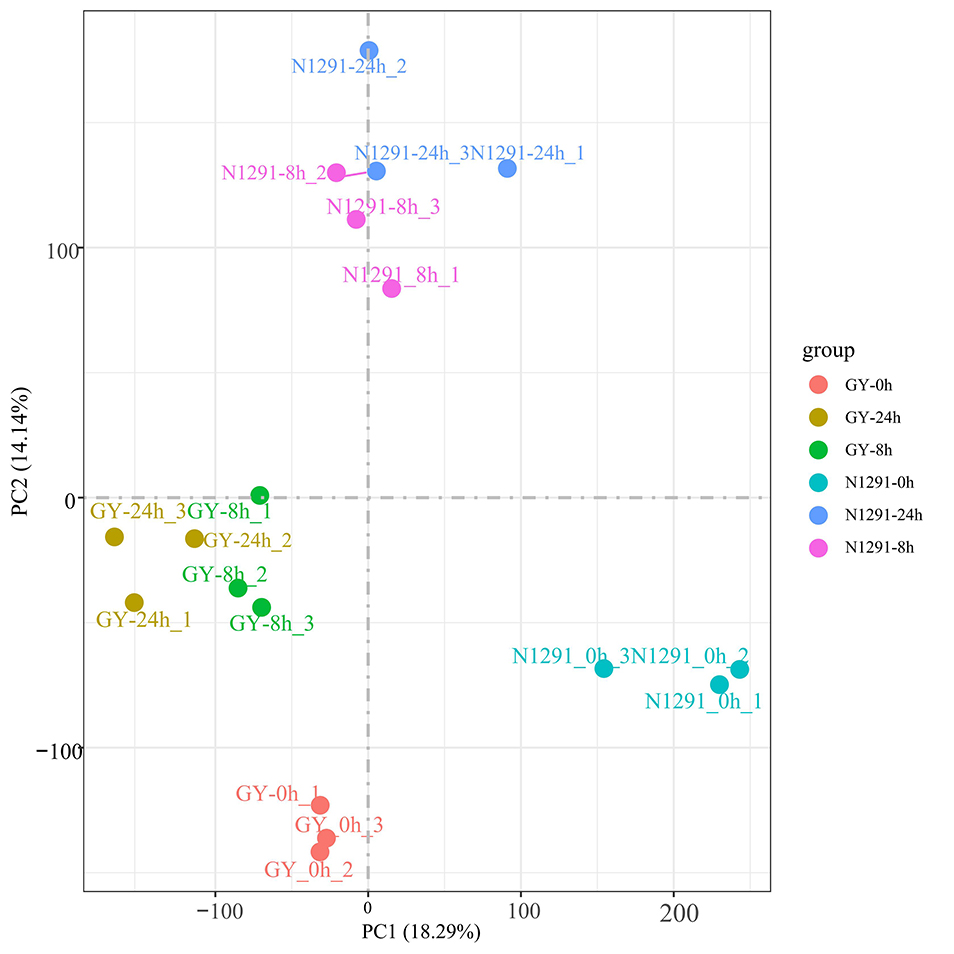

Supplement: Supplementary file 8 [file Image_1.jpeg]

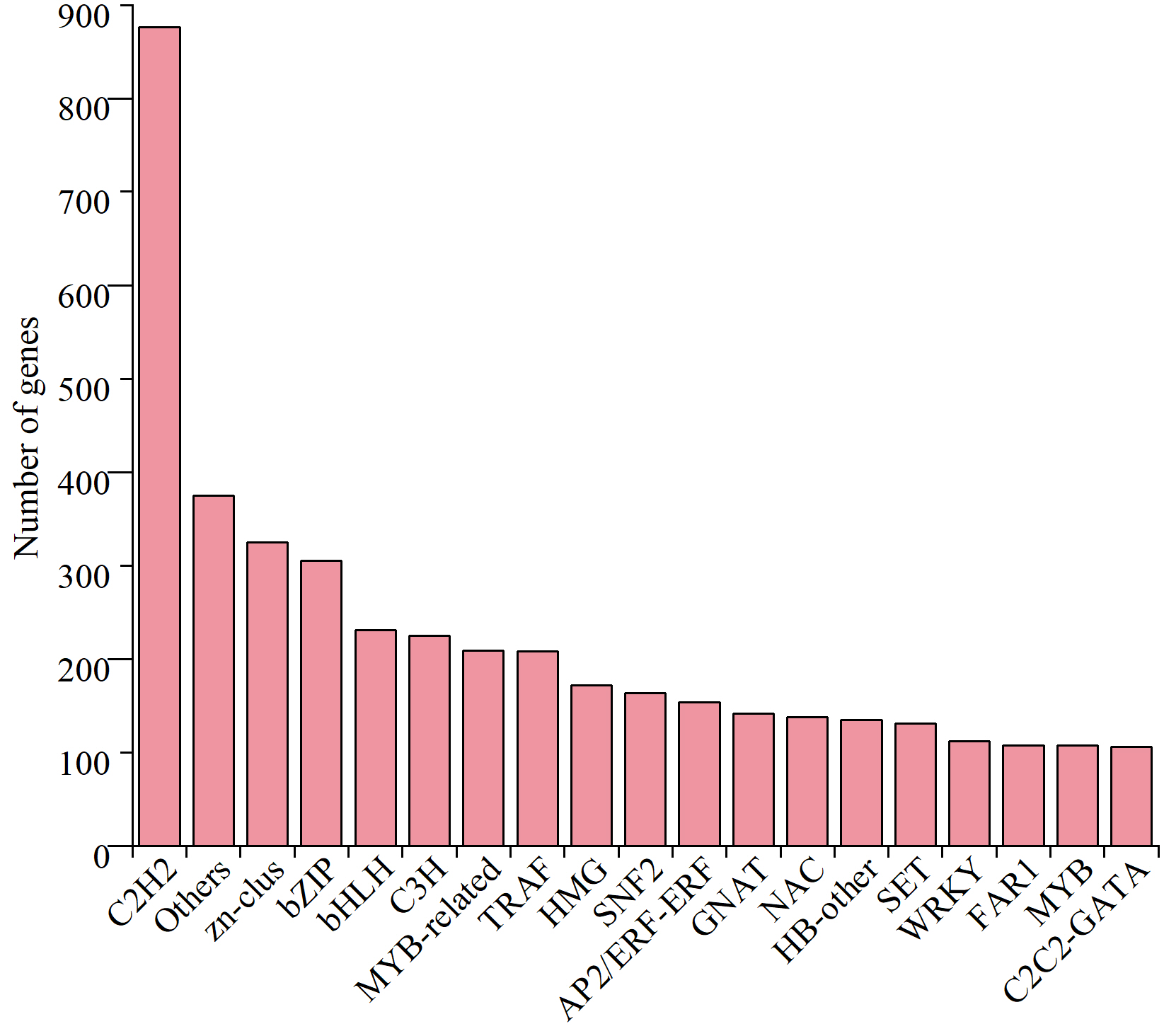

Supplement: Supplementary file 9 [file Image_2.jpeg]
